# Screening and Characterization of Phenolic Compounds from Australian Grown Bananas and Their Antioxidant Capacity

**DOI:** 10.3390/antiox10101521

**Published:** 2021-09-25

**Authors:** Yasmeen M. Bashmil, Akhtar Ali, Amrit BK, Frank R. Dunshea, Hafiz A. R. Suleria

**Affiliations:** 1 Department of Food Science and Nutrition, Faculty of Human Sciences and Design, King Abdulaziz University, Jeddah 21589, Saudi Arabia; ybashmil@student.unimelb.edu.au; 2 School of Agriculture and Food, Faculty of Veterinary and Agricultural Sciences, The University of Melbourne, Parkville, VIC 3010, Australia; akali@student.unimelb.edu.au (A.A.); amritb@student.unimelb.edu.au (A.B.); fdunshea@unimelb.edu.au (F.R.D.); 3 Faculty of Biological Sciences, University of Leeds, Leeds LS2 9JT, UK

**Keywords:** banana, polyphenols, antioxidant activities, characterization, identification, quantification, HPLC-PDA, LC-MS/MS

## Abstract

Bananas are an essential source of staple food and fruit worldwide and are widely regarded as the world’s largest fruit crop, with more than 100 million tons total annual production. Banana peel, a by-product that represents about 40% of the entire banana’s weight, and pulp are rich in bioactive compounds and have a high antioxidant capacity. As the production of polyphenols in fruit and vegetables is highly dependent on environmental conditions, genetic factors, and the level of maturity, this study aims to characterize six Australian banana cultivars in various stages of ripening for their phenolic compounds using the liquid chromatography-electrospray ionization quadrupole time of flight mass spectrometry (LC-ESI-QTOF-MS/MS), polyphenols quantification with the high-performance liquid chromatography coupled with photodiode array detector (HPLC-PDA), and their antioxidant capacity. All bananas were analysed for total polyphenols content (TPC), total flavonoids content (TFC), and total tannin content (TTC) and their antioxidant activities. Ripe Ducasse peel and pulp contained the highest amounts of total polyphenols content (1.32 and 1.28 mg gallic acid equivalent (GAE) per gram of sample), total tannin contents (3.34 mg catechin equivalent (CE) per gram of sample), and free radical scavenging capacity (106.67 mg ascorbic acid equivalent (AAE) per g of sample). In contrast, ripe Plantain peel had the greatest total flavonoids (0.03 mg quercetin equivalent (QE) per g of sample). On the other hand, unripe Ladyfinger pulp possessed the highest total antioxidant activity (1.03 mg AAE/g of sample). There was a positive correlation between flavonoids and antioxidant activities. By using LC-ESI-QTOF-MS/MS, a total of 24 phenolic compounds were tentatively characterized in this research, including six phenolic acids, 13 flavonoids, and five other polyphenols. Quantification of phenolic compounds by the high-performance liquid chromatography coupled with photodiode array detector (HPLC-PDA) revealed a higher content of phenolic acids. These findings confirmed that banana peel and pulp have considerable antioxidant activity and can be employed in human food and animal feed for variant health enhancement uses.

## 1. Introduction

Banana is a tropical seasonal fruit that encompasses a diverse range of species belonging to the genus *Musa* of the *Musaceae* family. It is among the world’s most popular fruits and the 4th most cultivated crop on a worldwide scale [1]. Almost all recognized cultivars are descended from two diploid species; *Musa balbisiana* and *Musa acuminata*, the most widespread of which are the Cavendish type [2]. Asia is the world’s largest banana producer, accounting for 54.4 percent of global banana production, as per the recent Food and Agriculture Organization (FAO) statistics. With an average usage of 12 kg per person per annum [3], bananas are one of the world’s primary food crops behind rice, wheat, and maize. In Australia, particularly in Queensland, banana production is one of the largest agricultural industries, with over 390,000 tonnes in 2015 [3] and around 600 merchant banana suppliers. The region of Australian banana cultivation varied between 10,000 and 16,000 ha during the period 1997–2015 [4]. Overall, 95% of banana genome groups in Australia are Cavendish, the remaining are Lady Fingers (AAB), Goldfingers (AAAB), Ducasse (ABB), Red Dacca (AAA), Sucrier (AA), and the Pacific Plantain (AAB) sub-group, and 95% of production is in North Queensland (QLD) [5].

The banana fruit is divided into two parts: the peel and the pulp. The peel, which is the fruit’s primary secondary product, accounts for around 40% of the fruit’s overall mass. Banana peels served no purpose and were discarded as waste, resulting in vast quantities of organic materials going into landfill. The central part, the pulp, the consumable portion of the fruit, is highly nutritious. Various research on banana pulp has examined various features, from its usage as a food fortification component to the extraction and recovery of numerous healthy constituents, including multiple kinds of starch, cellulose, and bioactive phytochemicals [6]. It is well established that banana flesh and skin comprise several secondary metabolites, such as catecholamines [7], phenolics [8], and carotenoids [9]. These bioactive substances promote probiotic development and help to reduce the risk of cancer and cardiovascular disease [10]. Pulp and peel parts include phenolic compounds, carotenoids, flavonoids, biogenic amines, phytosterols, and other phytochemicals [11]. Bananas have a greater antioxidant potential than various berries, herbs, and vegetables, attributed to the prevalence of these components [12]. Amongst the existing carotenoids in banana fruit, α-carotene, β-carotene, and β-cryptoxanthin have provitamin A activity, whereas others such as lycopene and lutein have a significant antioxidant capability [13]. Later, 171 various cultivars of *Musa* spp. were analysed for provitamin A carotenoids and 47 cultivars for two minerals, iron and zinc. It has been found that there is a great variability in provitamin A amongst the various genotypes, but a low variability in iron and zinc, regardless of the soil type and growing environmental conditions [14]. Moreover, banana pulp includes numerous antioxidants, such as phenolic compounds and vitamins, for example, catechin, epicatechin, lignin and tannin, and anthocyanin [15]. The catechins (epicatechin and gallocatechin) are most prevalent in the pulp when analysed by high-performance liquid chromatography [16]. Russel et al. have detected many phenolics in banana, such as ferulic, sinapic, salicylic, gallic, *p*-hydroxybenzoic, vanillic, syringic, gentisic, and *p*-coumaric acids as major components. Nevertheless, ferulic acid concentration was the highest among other phenolics [17]. According to Tsamo et al., hydroxycinnamic derivatives, such as ferulic acid-hexoside, were the major ones (4.4–85.1 µg/g DW) in plantain pulp. They discovered large differences in the phenolic contents among the tested varieties. In the plantain peels, rutin was the most abundant flavonol glycoside (242.2–618.7 µg/g DW). Therefore, the banana peel and pulp both are good sources of health-promoting phenolic compounds [18]. The main flavonoids detected in bananas are as follows: quercetin, myricetin, kaempferol, and cyanidin, which provide health benefits mostly due to their action as free radical scavengers [19]. Most of these phenolics, as natural antioxidants, have several biological effects, containing antiviral, antibacterial, antiallergenic, anti-inflammatory, vasodilatory and antithrombotic functions [20,21].

Although the concentration of phenolics in different banana cultivars has previously been investigated, a thorough profile of these compounds, especially in cultivars grown in Australia, remains inadequate due to their complicated composition and nature. Chemical analysis of these phenolics is typically performed by liquid chromatography-mass spectrometry (LC-MS) following sample extraction and filtering. Mass spectrometry is a well-established analytical technique used to infer unknown chemicals from complicated samples of various plant components, including banana [22].

To accomplish the study’s purpose, the polyphenols in six Australian grown banana cultivars, Cavendish, Ladyfinger, Red Dacca, Plantain, Monkey, and Ducasse were defined by TPC, TFC and TTC. In addition, the antioxidant capacity of all varieties was evaluated by ferric reducing antioxidant power (FRAP), 2,2′-diphenyl-1-picrylhydrazyl (DPPH), 2,2′-azinobis-(3-ethylbenzothiazoline-6-sulfonic acid) assay (ABTS), ferrous ion chelating assay (FICA), reducing power assay (RPA), hydroxyl radical scavenging assay (^•^OH-RSA) and total antioxidant capacity assay (TAC). Furthermore, the characterization and classification of phenolics were conducted using LC-ESI-QTOF-MS/MS, whereas the quantification of selected phenolics from various banana cultivars conducted using HPLC-PDA. As banana peel and pulp have a high antioxidant capacity, this study reinforces their usage as an origin of polyphenols in various industries, such as food, nutraceuticals, pharmaceuticals, cosmetics, and animal feed.

## 2. Materials and Methods

### 2.1. Chemicals and Reagents

Analytical grade reagents were utilized to extract and characterize banana. Sigma-Aldrich (Castle Hill, NSW, Australia) provided most of the chemicals used in the extracting and identification processes. Folin–Ciocalteu’s reagent, l-ascorbic acid, gallic acid, vanillin, hexahydrate aluminium chloride, hexahydrate iron [III] chloride (FeCl_3_ · 6H_2_O), sodium phosphate, sodium phosphate monobasic monohydrate, sodium phosphate dibasic hepta-hydrate, hydrated sodium acetate, trichloroacetic acid, ethylenediaminetetraacetic acid (EDTA), hydrochloric acid, ferrozine, iron (II) chloride, iron (III) chloride, ammonium molybdate, quercetin, 3-hydrobenzoic acid, catechin, iron (II) sulphate heptahydrate, potassium ferrocyanide (III), 2,2′-diphenyl-1-picrylhydrazyl (DPPH), 2,4,6tripyridyl-s-triazine (TPTZ), and ABTS, which used to estimate polyphenols and antioxidants capacity were bought from Sigma Aldrich (Castle Hill, NSW, Australia). Chem-Supply Pty Ltd. (Adelaide, SA, Australia) supplied sodium carbonate anhydrous and 30% hydrogen peroxide, while RCI Labscan (Rongmuang, Thailand) provided 98 percent sulfuric acid. Fifteen pure phenolic compounds (HPLC standards, 98–99.9%) including gallic acid, *p*-hydroxybenzoic acid, caftaric acid, caffeic acid, protocatechuic acid, chlorogenic acid, syringic acid, *p*-coumaric acid, catechin, Epicatechin, quercetin, quercetin-3-rhamnoside, diosmin, polydatin and kaempferol were produced by Sigma-Aldrich (Castle Hill, NSW, Australia) for quantification proposes. Thermo Fisher Scientific Inc. (Scoresby, VIC, Australia) supplied LC-MS and HPLC grade chemicals such as ethanol, methanol, formic acid, acetonitrile, iron (III) chloride anhydrous, and glacial acetic acid. Then, 96-well plates were acquired from Thermo Fisher Scientific (Scoresby, VIC, Australia) to conduct several biological activities and antioxidant experiments. Furthermore, Agilent technologies (Melbourne, VIC, Australia) provided HPLC 1 mL vials.

### 2.2. Phenolic Compounds’ Preparation and Extraction

**Figure 1:**
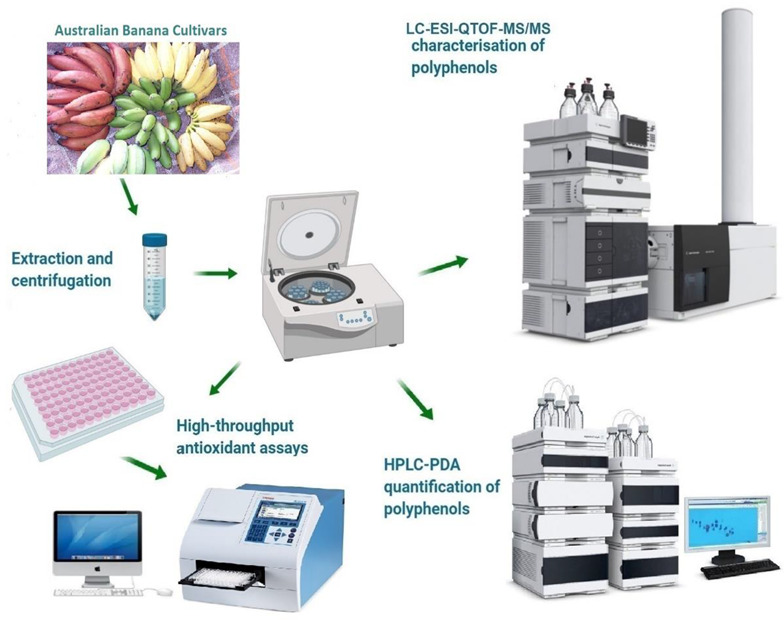
Graphical abstract and schematic layout of this study.

Six banana cultivars (Cavendish, Ladyfinger, Plantain, Ducasse, Monkey, and Red dacca) were purchased from different local markets in Melbourne. Two ripening stages were chosen for this study, all green and all yellow, which are stages 1 and 6 according to Soltani, Alimardani, and Omid [23]. Each part (peel and pulp) was ground separately into small pieces with a grinder. The approach of Peng, et al. [24] was used and modified to extract phenolic components from various banana samples. To summarize, the extracts were produced by diluting the ethanol with Milli-Q water to get 70% concentration, with the addition of potassium metabisulfite to stop enzymatic browning reactions [25]. After administering 30 mL solvent to a 10 g sample in triplicate, they were put in a shaker (ZWYR-240 incubator shaker, Labwit, Ashwood, VIC, Australia) for 16 h at a temperature of 20 °C and a speed of 150 rpm to allow for probable phenolic compound separation. Following that, all samples were centrifuged for 15 min at 8000 rpm (ROTINA380R, Hettich Refrigerated Centrifuge, Tuttlingen, Baden-Württemberg, Germany). The supernatant was taken and kept at a temperature of 20 °C to maximise the antioxidant activity. The extracts were filtered in HPLC vials using a syringe filter for LC/MS analysis. Figure 1 shows the graphical abstract of this study.

### 2.3. Antioxidant Experiments

Antioxidant assays were done in accordance with the method of Ali et al. [22] and Gu et al. [26] and all experiments were conducted in triplicate in 96-well plates. Standard curves were created and samples analysed by recording the absorbance with a Multiskan microplate photometer.

#### 2.3.1. Estimation of Total Polyphenols Content (TPC)

The TPC of all banana extracts was evaluated using a modified method of Vallverdú-Queralt, et al. [27]. To begin, 25 μL (25 percent Folin–Ciocalteu reagent *v/v*) was mixed with 25 μL of samples in 96-well plates together with 200 μL MilliQ water. The plate was then incubated at 25 °C for 5 min. After that, 25 μL (10% *v/v* sodium carbonate) was added to the mixture and left in a dark place for 60 min at 25 °C. The absorbance at 765 nm was observed. The TPC was determined using a standard curve constructed for gallic acid concentrations ranging from 0–200 μg per mL in ethanol. The TPC was expressed in milligram gallic acid equivalents (GAE) per gram fresh weight of samples.

#### 2.3.2. Estimation of Flavonoids Content (TFC)

The TFC were evaluated by the AlCl_3_ colorimetric technique of Muhammad, et al. [28] after adding some modifications. In 96-well plates, 80 μL of sample extracts were combined with 80 μL of 2% aluminium chloride solution and 120 μL sodium acetate solution. This mixture was kept at 25 °C in the dark for 2.5 h, and at 440 nm, absorbance was measured. All samples were measured in triplicate, and TFC was determined using a standard curve constructed for 0–50 μg per mL quercetin. The data were represented as (mg) quercetin equivalents (QE) per (g) fresh weight of the samples (*r*^2^ = 0.999).

#### 2.3.3. Estimation of Total Tannins Content (TTC)

The TTC in banana was evaluated following the method described by Feng, et al. [29] with some adjustments by the 96-well plate technique. This was accomplished by mixing 25 µL of sample solution was mixed in a 96-well plate with 150 µL of 4% vanillin solution. Then, 25 µL of 32% H_2_SO_4_ was blended with this mixture and incubated at 25 °C for 15 min, then at 500 nm, the absorbance was determined. All samples were quantified in triplicate using a standard curve of catechin in methanol 0–1000 µg/mL. The results were given as mg CE per gram fresh weight of the samples.

#### 2.3.4. 2,2′-Diphenyl-1-Picrylhydrazyl Assay (DPPH)

The DPPH assay was used to estimate the free radical scavenging capacity of all banana samples by modifying the method of Sokamte, et al. [30]. Briefly, a 25 μL extract of each sample was mixed with 275 μL DPPH solution made 0.1 M in methanol and placed for 30 min at 25 °C in dark. After that, absorbance was recorded at 517 nm and the results were expressed as mg AAE/g. The standard curve was generated by using the ascorbic acid 0–50 μg/mL in water.

#### 2.3.5. Ferric Reducing Antioxidant Power Assay (FRAP)

FRAP activity was estimated by following Chen, et al. [31] and Sharifi-Rad, et al. [32] with some modifications. To do this, sodium acetate buffer (300 mM), TPTZ (10 mM) and ferric chloride (20 mM) were mixed in the ratio of 10:1:1 (*v:v:v*) to make FRAP reagent. Then, 20 μL of banana sample extract and 280 μL FRAP reagent were mixed together and placed the reaction mixture at 37 °C for 10 min. After that, absorbance was recorded at 593 nm and the results were expressed as mg AAE/g. The standard curve was generated by using the ascorbic acid 0–50 μg/mL in water.

#### 2.3.6. 2,2′-Azinobis-(3-Ethylbenzothiazoline-6-Sulfonic Acid) (ABTS) Assay

The ABTS radical scavenging ability of all the samples was determined using a modified method of Severo, et al. [33]. This was achieved by mixing 7 mM ABTS solution with 140 mM potassium persulfate solution. To form an ABTS solution, the mixture was incubated in a dark place for 16 h. At 734 nm, this solution was diluted with ethanol to achieve an absorbance of 0.70 ± 0.02 at that wavelength. Following that, 10 µL of extract was combined with 290 µL of ABTS solution in 96-well plate inculpated at 25 °C for 6 min in a dark room. The absorbance at 734 nm was then monitored. The quantification was performed by creating a standard curve versus ascorbic acid concentrations ranging from 0–150 µg per mL in water. The resulted data were presented as milligram AAE/g.

#### 2.3.7. Reducing Power Assay (RPA)

The reducing power capacity was assessed using an adjusted approach of Ferreira, et al. [34]. A total of 10 μL of sample extract was mixed with 25 μL of 0.2 M phosphate buffer (pH 6.6), and 25 μL of potassium ferricyanide, followed by a twenty-minute incubation at 25 °C. The reaction was then terminated with 25 μL of 10% TCA solution followed by addition of water (85 μL) and 8.5 μL of FeCl_3_ and incubation for an additional 15 min at 25 °C. Following that, the absorbance at 750 nm was determined. A standard curve was constructed using ascorbic acid concentrations ranging from 0–300 μg per mL and findings were presented in milligram AAE per gram.

#### 2.3.8. (^•^OH) Radical Scavenging Assay

To determine ^•^OH radical scavenging ability, the technique of Smirnoff and Cumbes [35] was used with some adjustments. A 50 μL extract of the sample was combined with 50 μL of 6 mM iron(II) sulphate hydrate and 50 μL of 6 mM hydrogen peroxide (30%), then incubated for 10 min at 25 °C. Following that, 50 μL of 6 mM 3-hydrooxybenzoic acid was added, and the absorbance at 510 nm wavelength was recorded. A standard curve was constructed using ascorbic acid concentrations ranging from 0–300 μg per mL and findings presented in mg AAE per g.

#### 2.3.9. Determination of Ferrous Ion Chelation Activity (FICA)

Banana samples’ Fe^2+^ chelation potential was detected using the method of Dinis, et al. [36] after modification. A 15 μL extract of sample was combined with water (85 μL), 50 μL of 2 mM FeCl_2_ (with further dilution 1:15 in water) and 50 μL of 5 mM ferrozine (which diluted further in water 1:6), followed by a 10 min incubation at 25 °C. The absorbance was then determined at 562 nm wavelength. A standard curve was constructed using EDTA at values ranged from 0–50 μg per mL, and values were expressed as mg EDTA/g.

#### 2.3.10. Total Antioxidant Capacity Assay (TAC)

The TAC of all banana extracts was determined using the approach of Nićiforović, et al. [37] with some changes. This was accomplished by adding 40 μL of every banana sample to 260 μL of phosphomolybdate reagent (0.6 M H_2_SO_4_, 0.028 M sodium phosphate and 0.004 M ammonium molybdate). After incubation for 90 min at 95 °C, the mixture was cooled to room temperature. The absorbance was recorded at 695 nm. A standard curve was created using ascorbic acid concentrations ranging from 0 to 200 μg/mL, and the findings were presented as mg AAE/g.

### 2.4. Identification of Phenolic Compounds from Banana Extracts by LC-ESI-QTOF-MS/MS

The characterization of polyphenols from all banana extracts was done using the method of Suleria, et al. [38] with some changes. To achieve this, the phenolic compounds were characterized using an Agilent 1200 HPLC in conjunction with an Agilent 6520 Accurate Mass Q-TOF- LC-MS/MS (Agilent Technologies, Santa Clara, CA, USA). The isolation was carried out by a Synergi Hydro-RP 80 Å, and a reverse-phase column (250 mm × 4.6 mm, 4 μm particle size) (Phenomenex, Lane Cove, NSW, Australia). Briefly, the mobile phase was composed of (eluent A) water and formic acid (99.9:0.1, *v/v*), besides (eluent B) acetonitrile, water and formic acid (95:5:0.1). Then, 6 µL of every banana extract was administered at a flow average of 600 μL/minute. Peaks were detected in negative and positive ion modes. Moreover, the following parameters were preserved; temperature of nitrogen gas at 325 °C, 5 L/min flow average of sheath gas at 325 °C, nebulization of nitrogen gas at 30 psi. A range of *m/z* 50 to 900 was utilized to obtain complete mass scan. For fragmentation, collision energies (10, 15, and 30 eV) were used, and MS/MS investigation was performed in automated mode. The machine control, data acquisition, and analysis were conducted by LC-ESI-QTOF-MS/MS Mass Hunter workstation software (Qualitative Analysis, version B.06.01, Agilent Technologies, Santa Clara, CA, USA).

### 2.5. Quantification of Bananas’ Phenolics by HPLC-PDA

The method of Tang, et al. [39] and Chou, et al. [40] with some changes was used to quantify polyphenols in banana extracts. This was accomplished using a Water Alliance (2690) HPLC coupled with photo array detector (PDA). The same LC/MS column was used as described previously. In summary, the mobile phase was composed of (eluent A) water and acetic acid (98:2, *v/v*) and (eluent B) acetonitrile, water, and acetic acid (50:49.5:0.5). The temperature of samples and column was maintained at room temperature throughout the chromatographic process. As 20 µL extract of every sample was injected and the flow rate averaged 800 μL/min. Fifteen phenolic compounds (gallic acid, *p*-hydroxybenzoic acid, caftaric acid, caffeic acid, protocatechuic acid, chlorogenic acid, syringic acid, *p*-coumaric acid, catechin, Epicatechin, quercetin, quer-cetin-3-rhamnoside, diosmin, polydatin and kaempferol) were identified and quantified in triplicate at three different wavelengths, including 280 nm, 320 nm and 370 nm. The quantification of the concentration of individual polyphenols was based on the calibration standard curve (0–250 ug/mL) and the result was expressed as mg/g of fresh sample.

### 2.6. Statistical Analysis

Samples’ phenolic content and antioxidant experiments data were expressed as the means ± standard deviation, and one-way analysis of variance (ANOVA) was used to determine whether there were significant differences in mean values between different samples, followed by Tukey’s honestly significant differences (HSD) multiple rank test at *p* < 0.05. Minitab program 18.0 for Windows was used to conduct ANOVA test. XLSTAT-2019.1.3 (Addinsoft Inc. New York, NY, USA) was used to study the correlation between polyphenols and antioxidant capacity.

## 3. Results and Discussion

Banana polyphenol identification, characterization, and validation of banana polyphenols were accomplished using LC-ESI-QTOF-MS/MS, and quantification was completed by using HPLC-PDA. A strong positive correlation was found between phenolics and their antioxidant capacity.

### 3.1. Polyphenols Assessment of Banana

The assessment of polyphenols in various banana cultivars was attained via TPC, TFC, and TTC. Diets high in fruits and vegetables have been associated with a decreased risk of heart diseases and cancer. The antioxidant components of fruit and vegetables, for example, phenolic acids, flavonoids, tannins, lignans and stilbenes with pharmacological and biological attributes, have been credited with their protecting impact [41]. Multiple factors, such as plants’ varieties, degree of ripening, growing environment, and initial treatments, can affect the composition and concentrations of these components [42].

The highest phenolic contents were found in the ripe Ducasse peel and pulp, unripe Ladyfinger peel, ripe Plantain peel, unripe Monkey peel and unripe Ladyfinger pulp, which contained 1.32, 1.28, 1.15, 0.87, 0.79 and 0.76 mg GAE/g, respectively, whereas the lowest value of TPC was found in ripe Red Dacca pulp (0.40 mg GAE/g) and ripe Plantain pulp (0.38 mg GAE/g) (Table 1).

TPC values ranging from 0.15 to 55.5 mg GAE/g had already been reported in twenty-seven different banana cultivars in which the Brazilian Cavendish has the second highest value (29.2 mg GAE/g) [42]. However, TPC differences can be ascribed to extraction circumstances (solvent kind, concentration, solvent/sample ratio, and time/temperature combination for extracting), cultivar variations, and the geographical location of where the bananas were grown [43]. Flavonoids are the most abundant class of phenolic compounds present in practically all plants, as shown in this study using the aluminium chloride colorimetric method.

Aluminium chloride interacts with the flavonoids’ carbonyl group, generating a stable compound [44]. The TFC values in the banana samples had a similar pattern of TPC results, in which the most prevalent flavonoid compounds were identified in the ripe Ducasse pulp (0.03 mg QE/g), and peel (0.02 mg QE/g), whereas the lowest values were found in ripe Monkey peel (0.006 mg QE/g). TFC values ranged from 8.56 ± 0.22 to 16.15 ± 0.28 mg QE/g in Egyptian banana cultivars, according to Aboul-Enein, et al. [45], which was higher than our Australian samples. This may be due to varieties divergence or type of solvent used for the extraction of phenolic compounds. Comparing to other tropical fruits, it has been found that the mango peel showed the maximum concentration of flavonoid (1.75 ± 0.08 mg QE/g), then pineapple and banana peels (1.47 ± 0.07 and 1.32 ± 0.12 mg QE/g), respectively [38].

Tannins are also a substantial family of phenolic substances that could be partitioned into hydrolysable and non-hydrolysable tannins. In our banana samples, the ripe Ducasse peel had the highest amount (3.34 ± 0.2 mg CE/g), followed by the unripe Ladyfinger pulp (1.52 ± 0.09 mg CE/g) and unripe Cavendish pulp (0.66 ± 0.08 mg CE/g). Most fruits show a reduction in tannins content when they ripen, which is believed to be due to tannin polymerisation [46,47,48]. According to Von Loesecke [49], several forms of tannin and soluble tannin become insoluble throughout ripening. Barnell and Barnell [46] explained that the Latex vessels in both the pulp and peel and small dispersed cells in the peel are two kinds of tannin-containing elements. Furthermore, they suggested that the tannin structure in latex vessels varies, whereas the tannin content of the peel’s tiny dispersed cells tends to have little or no alteration. On the other hand, Kyamuhangire et al. [50] found that Mbidde bananas had a greater tannin content than Matooke bananas at both the green and mature phases. The high tannin content is due to the unusual presence of laticifer cells in the fruit of Mbidde varieties, which is similar to our findings in ripe Ducasse peel. Moreover, their results showed that the amount of extractable tannins improved with ripening in all four banana cultivars, and after ripening, the amount of water-extractable tannins increased significantly in Mbidde bananas, but a very small amount was found in Matooke bananas. The increased amount of extractable tannins appears to indicate that tannins become more accessible as the banana ripens. This contrasts with the observation that bananas lose their astringent flavour during maturation, a phenomenon explained by increasing tannin polymerisation [46] and inactivity [51].

### 3.2. Antioxidant Capacity of Banana

Polyphenols are essential antioxidants found in plants and have a variety of biological actions. They are proven to have several functions because they work in biological systems as reducing chemicals, metal chelators, hydrogen atom donors, scavengers of radical oxygen. The antioxidant capacity of banana was further studied using a variety of methods, including radical scavenging ability and reducing power of the sample. For this aim, DPPH, FRAP, ABTS, RPA, FICA, ^•^OH-RSA and TAC experiments were performed, and the results presented in Table 2.

The DPPH, FICA, ABTS, and ^•^OH-RSA tests have been primarily utilized to assess the scavenging activity of bioactive metabolites, primarily polyphenols [52]. In contrast, the FRAP assay assesses samples’ ability of electrons donation to convert a ferric-TPTZ complex to a blue ferrous-TPTZ complex. DPPH can supply hydrogen ions to the biological system or act as a scavenger of free radicals. When DPPH is combined with banana samples, it accepts hydrogen atoms, and diminishing violet colour occurs. The DPPH, ABTS, and FRAP values of ripe Ducasse pulp and ripe Ladyfinger peel (1.68 mg AAE/g, 2.69 mg AAE/g, and 2.85 mg AAE/g, respectively) were considerably greater than those of other listed cultivars. In contrast, ripe Plantain pulp, ripe Red Dacca pulp, and ripe Monkey peel had the lowest DPPH, FRAP, and ABTS values (0.069 mg AAE/g, 0.36 mg AAE/g and 0.59 mg AAE/g), respectively.

The DPPH values of 23 variant banana cultivars grown in different countries, ranging from 0.68 to 66.9 mg AAE/g, were recorded in a previous review, and our findings were close to some of those varieties [42]. The ability of banana peels to scavenge free radicals (DPPH) decreases as the fruit progresses from green to ripe to overripe [53,54]. Nevertheless, the electron transfer/hydrogen donating capacity of phenolics’ nature is also recognized to be connected to their DPPH^•^ radical scavenging activity. Numerous substances of botanical origin have an antioxidant activity proportional to their phenolic content, indicating a causative correlation between total phenolic concentration and antioxidant activity [55]. Furthermore, our banana FRAP values were within the range of other banana cultivars studied (0.32–17.64 mg AAE/g) by Vu, Scarlett and Vuong [42]. The FRAP value of the Brazilian Cavendish peel, which is yellow with a green edge, was higher (14 mg AAE/g) [56]. Guo [50] mentioned that most fruit peels demonstrated 2- to 27-fold greater antioxidant activity than the fruit flesh, and variations between varieties can occur because the antioxidant activity of fruits can be influenced by geographical location, variety, and harvesting or storing period. Furthermore, it is well established that phenolics’ antioxidant capacity depends on their molecular weight, the amount of aromatic rings, and the number and position of hydroxyl groups attached to aromatic ring [57]. Additionally, it has been claimed that parameters such as the stereoselectivity of the radicals or the solubility of phenolics in various testing conditions influence the capacity of extracts to react with and eliminate various radicals [58]. According to Hagerman, et al. [59], tannins, as high molecular weight phenolics, have a greater capacity to scavenge free radicals (ABTS^•+^).

Regarding RPA, ^•^OH-RSA and FICA assays, Ladyfinger, Monkey, and Ducasse peels had higher antioxidant capacity than other varieties. In RPA, ripe Ladyfinger peel had the highest antioxidant potential, followed by ripe Ducasse pulp and unripe Ladyfinger peel. In FICA assay, Ripe Monkey peel had high antioxidant activity compared to other cultivars. On the other hand, ripe Ducasse peel had higher ^•^OH-RSA value (106.67 mg AAE/g), followed by unripe Cavendish peel (105.95 mg AAE/g). To our best knowledge, this is the first time that banana’s antioxidant potential has been analysed through the RPA, ^•^OH-RSA and FICA assays. Previously, a thorough phenolic analysis of custard apples cultivated in Australia has been performed by Du et al. [60]. They found that African Pride peel possessed the greatest antioxidant capacity for RPA (5.32 ± 0.14 mg AAE/g), ^•^OH-RSA (1.23 ± 0.25 mg AAE/g) and FICA (3.17 ± 0.18 mg EDTA/g). Based on the current iron chelating findings, the extracts may be able to protect against oxidative stress by isolating Fe(II) ions that would otherwise accelerate Fenton-type reactions or engage in metal catalysed hydroperoxide breakdown reactions. The samples’ Fe(II) chelating properties may be a result of their endogenous chelating compounds, primarily phenolics [61]. Grinberg et al. [62] claimed that tea polyphenols’ protective action against OH-dependent salicylate hydroxylation was due to iron chelation. The research on polyphenols’ deleterious effects on iron bioavailability has emphasized that iron binding to flavonoid antioxidants can diminish iron’s accessibility to oxygen molecules. In contrast, non-flavonoid polyphenols can reduce Fe^3+^ ions and then create inert Fe^2+^ polyphenol complexes [63].

In the TAC assay, which was conducted by reducing molybdenum (VI) to molybdenum (V) in the presence of phenolics, the pulps of the banana varieties had generally higher values than peels. The highest value was observed in unripe Ladyfinger pulp (1.03 ± 0.08 mg AAE/g), followed by ripe Ducasse pulp (10.43 ± 0.20 mg AAE/g) and unripe Cavendish pulp (0.58 ± 0.03 mg AAE/g).

Remarkably, the antioxidant activity of fruits varies depending on the extraction method that was utilized. Fruit extracts contain a diverse array of bioactive chemicals. The amount of phenolic acids and flavonoids in each type is highly variable and is influenced by cultivar, geographic location, and climates. There are several procedures for determining the antioxidant capacity, and each methodology has several advantages and disadvantages. To summarize, our findings indicate that each banana cultivar’s antioxidant activity varies according to its phenolic profile or the method utilized to quantify it. Numerous in vitro experiments may be used to determine the antioxidant capacity of banana, while modern analytical techniques like as LC-ESI-QTOF-MS/MS can be used to identify and confirm these antioxidant components.

Phenolics are a broad collection of molecules that are garnering attention in research due to their possible health benefits. Due to the antioxidant properties of polyphenols, they may be used to prolong the shelf life of lipid-rich products. Additionally, bananas have diverse antibacterial phenolic compounds that act as preservatives for food during storage. Furthermore, because phytochemicals have a complex structure, no one approach accurately evaluates polyphenols’ antioxidant capacity due to the various pathways and reactions occurring in the biological system. Thus, LC-MS characterization is one of the sophisticated research approaches used to profile polyphenols and better understand the banana’s total antioxidant capacity. The polyphenol contents of banana and their antioxidant capacity indicate that additional research should be undertaken to determine the true contribution of phenolics to the antioxidant potential while excluding or limiting non-phenolic substances’ involvement.

### 3.3. Correlation between Polyphenols and Antioxidant Potentials

A regression analysis was used to determine the association between the findings of the experiments undertaken (Table 3).

Due to the critical antioxidant effects of polyphenols observed in vegetables and fruits, we studied TPC, TFC, and TTC in Australian bananas. TPC was shown to have a range of 0.40–1.32 mg GAE/g, with an average of 0.68 mg GAE/g (Table 1). The highest TPC value was found in Ducasse peel, while the least was reported in Red Dacca pulp. The antioxidant activity of banana extracts was determined using DPPH, FRAP, ABTS, RPA, ·OH-RSA, FICA, and TAC.

Between total phenolic content and the antioxidant activities of DPPH, FRAP, and RPA, ·OH-RSA, and TFC, a high and significant positive correlation was detected, but TFC was only correlated significantly with DPPH and FRAP. Previously, a positive correlation between antioxidant activity and total polyphenols in herbs and spices was discovered [64]. Notably, there was a negative association between TPC, TFC, and TTC and TAC, ABTS, and FICA.

TAC is also negatively correlated with ABTS, ·OH-RSA, RPA, and FICA. Total phenolics have been shown to be responsible for the antioxidant capacity of plant diets [65]. Numerous variables affect the correlation, including the number of samples analysed, the quantities measured, and the antioxidant assays used. DPPH and FRAP were highly correlated in our research. Kim, Yang, Lee and Kang discovered that TPC had a higher relationship with antioxidant activity than TFC [28].

**Figure 2:**
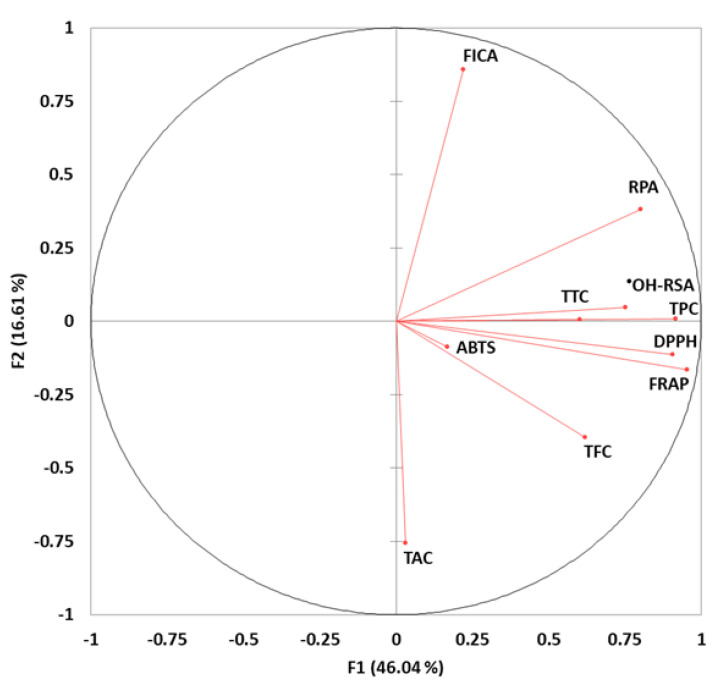
Principal component analysis (PCA) of the phenolic contents (TPC, TFC, TTC, phenolic acids, and flavonoids) and their antioxidant capacities (DPPH, FRAP, ABTS, RPA, ^•^OH-RSA, FICA, and TAC) of banana cultivars.

Principal component analysis (PCA, Figure 2) demonstrated that TPC and TTC were positively correlated with ˙OH-RSA, RPA, and FICA with higher scores. Furthermore, FRAP, TFC, DPPH, and ABTS were positively correlated, while FICA showed negative correlation with TAC, TFC, ABTS, FRAP and DPPH, which revealed the divergence of bioactive components in banana cultivars.

After evaluating the data, it is indicated that the antioxidant activity of banana extracts may also be related to non-phenolic elements. Even though simple phenols have relatively little contribution to antioxidant potentials, they can interact with Folin–Ciocalteu reagent [22]. The current study’s findings reveal that distinct phenolic compounds exhibit varying levels of antioxidant activity based on their structure, synergistic effect, quantity, and hostile attitude towards other components appearing in each banana extract.

### 3.4. LC-ESI-QTOF-MS/MS Identification of the Phenolic Compounds

To identify and characterize bioactive substances, such as phenolics from various fruits, vegetables, and herbal medicines, LC-MS/MS has been broadly used. A LC-ESI-QTOF-MS/MS analysis in negative and positive ionization modes was used to obtain a non-targeted qualitative analysis of phenolics from Australian banana cultivars pulp and peel samples (Table 4) utilizing Agilent LC-MS Qualitative Software and Personal Compound Database and Library, phenolics in banana samples were identified and based on their *m/z* value and MS spectra in both negative and positive modes of ionization ([M–H]^–^/[M+H]^+^). For further MS/MS detection, *(m/z*) characterization, and verification, compounds with a mass error of less than ±10 ppm were used. LC-MS/MS was used to identify 24 phenolic compounds in banana samples, which include phenolic acids (6), flavonoids (13), and other polyphenols (5) mentioned in Table 4.

#### 3.4.1. Phenolic Acids

Phenolic acids are phenolic compounds that are abundant in some banana varieties. Phenolic acids are prevalent in fruits and vegetables and have been extensively studied for their potential benefits, including antioxidant, antimicrobial, anti-inflammatory, antimutagenic properties, etc. [66]. This research found six phenolic acids, including one hydroxybenzoic acid, three hydroxycinnamic acids, one hydroxyphenylacetic acid, and one hydroxyphenylpropanoic acid (Table 4). The majority of phenolic acids displayed neutral loss of CO_2_ (44 Da) and hexosyl moiety (162 Da).

##### Hydroxyphenylpropanoic Acids

Compound 1 was identified by its mass spectrum as 4-hydroxyphenyl propionic acid, which was detected in ripe Cavendish peel. The main component at *m/z* 165 resulted from the loss of H from [M–H]^–^. Other important fragment ions identified were at *m/z* 121 and *m/z* 119, related to loss of CO_2_ and HCOOH, respectively [67].

##### Hydroxycinnamic Acids

Hydroxycinnamic acids were found in greater quantity than other phenolic acids in this study. Our analysis discovered a total of three hydroxycinnamic acids with strong antioxidant activity. Ferulic acid (Compound 2) was detected in ripe Cavendish pulp. In this MS/MS experiment, ferulic acid exhibited the product ions at *m/z* 178, *m/z* 149, and *m/z* 134, showing the loss of CH_3_, CO_2_, and CH_3_ with CO_2_ from the precursor, respectively [68]. Compound 3 *(m/z* 179.035) was identified in unripe Cavendish pulp, and exhibited a product ion at *m/z* 143 and 133 (caffeic acid ion) by glucoside loss (162 Da) in negative mode and presented as caffeic acid [68]. Compound 4 exhibits product ions at *m/z* 143 and *m/z* 133 with expected composition (C_11_H_10_O_5_), classified as *p*-Coumaroyl glycolic acid. Caffeic acid is a crucial step in the synthesis of lignin with potential antioxidant. This class of polyphenol derivatives has been implicated in the antioxidant activity of different fruit and vegetable varieties [69].

##### Hydroxybenzoic Acids

Hydroxybenzoic acids are abundant in a variety of fruits, including mango, apple, custard apple, citrus, strawberries, and raspberries, and have a high antioxidant capacity. Compound 6 (*m/z* 199.0595) was recognized as 3,4-*O*-Dimethylgallic acid, with product ions at *m/z* 153, 139, 125, and 111, indicating the loss of CO_2_ from the precursor ions [70]. Earlier, Kim [71] had also found gallic acid in the peel and pulp of white and red dragon fruit.

##### Other Phenolic Acids

Compound **5** (*m/z* 167.0341) was characterized as 3,4-Dihydroxyphenylacetic acid, which produced product ions at *m/z* 149 and *m/z* 123 with a unit of H_2_O (18 Da) and CO_2_ (44 Da), which was confirmed through MS/MS [38].

#### 3.4.2. Flavonoids

The most prevalent class of antioxidants detected in the banana samples was flavonoids (13 in total). Flavonoids were classified into six subclasses, (1) flavanols, (2) flavones, (1) flavanones, (4) flavonols, (4) anthocyanins and (1) isoflavonoids. One flavanone was found in banana, neoeriocitrin (Compound 19), which was identified in negative mode and recognized by the precursor ions at *m/z* 431 and *m/z* 287. The product ion at *m/z* 303 in the MS/MS spectrum of quercetin 3’-*O*-glucuronide, a flavonols, was formed by the removal of glucuronide (176 Da) from the precursor [56], and the peak at *m/z* 301 (loss of pentose moiety, 132 Da) proved the identity of myricetin 3-*O*-arabinoside [72]. Additionally, Compound 12, isorhamnetin 3-*O*-glucoside 7-*O*-rhamnoside, was classified as a flavonol by the precursor ion at *m/z* 623.1614 [M–H]^–^ (C_28_H_32_O_16_) [73].

### 3.5. Heatmap and Hierarchical Analysis of Bananas’ Polyphenols

**Figure 3:**
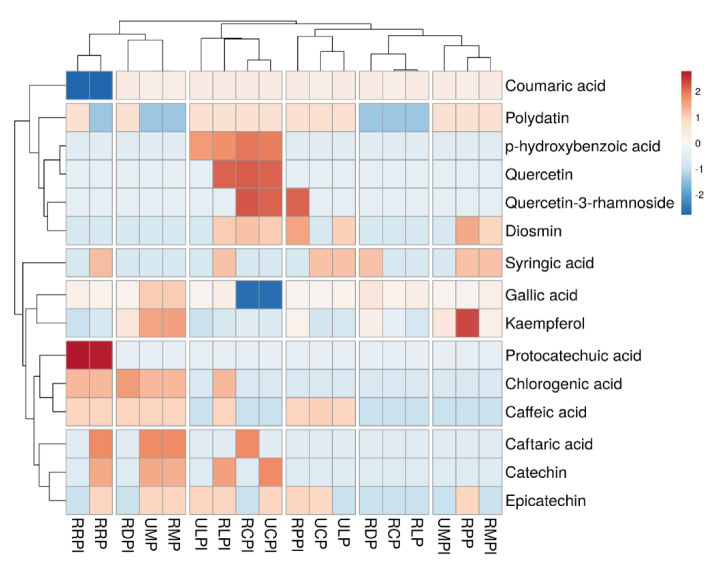
Heatmap showing “distribution and concentration” of quantified phenolic compounds (mg/g) in all banana samples. Red colour boxes indicating the higher concentration while blue colour boxes showing lower or zero concentration. Banana were presented with abbreviations. Ripe Cavendish peel (RCPl), Ripe Cavendish pulp (RCP), Unripe Cavendish pulp (UCP), Unripe Ladyfinger peel (ULPl), Ripe red dacca peel (RRPl), Ripe Plantain peel (RPPl), Ripe Monkey pulp (RMP), Ripe Ducasse peel (RDPl), Ripe Ladyfinger peel (RLPl), Ripe Ladyfinger pulp (RLP), Unripe Ladyfinger pulp (ULP), Unripe Monkey peel (UMPl), Unripe Monkey pulp (UMP), Ripe Monkey peel (RMPl), Ripe Plantain pulp (RPP), Ripe Ducasse pulp (RDP), Unripe Cavendish peel (UCPl), and Ripe red dacca pulp (RRP).

To aid in the subsequent analysis of HPLC-PDA data for 15 phenolic compounds isolated from various banana cultivars, a heatmap was created (Figure 3). Six clusters were formed in a row and column manner and emphasized using hierarchical grouping.

The variation in clustering reveals the difference in phenolic component concentrations in banana. The change in colour profile shows the presence of various phenolic acids and flavonoids. In general, ripe Ladyfinger peel contained more compounds, the majority of which were phenolic acids. The red colour boxes in ripe Ladyfinger peel (quercetin, *p*-hydroxybenzoic acid, catechin, chlorogenic acid, gallic acid, syringic acid, diosmin, and *p*-coumaric acid) indicate the presence of these substances in greater concentration than other components [74]. However, protocatechuic acid and quercetin-3-rhamnoside, were discovered with greater concentration in ripe Red Dacca peel and pulp, and unripe and ripe Cavendish peel. Moreover, kaempferol is one of the flavonols which was abundantly found in ripe plantain pulp and less or absent from other cultivars. In Cavendish peel and pulp, *p*-hydroxybenzoic acid, quercetin and quercetin-3-rhamnoside were found at high amounts, whereas *p*-coumaric acid, epicatechin, polydatin, and diosmin were less prevalent. In previous studies, HPLC analysis found seven phenolic compounds and three flavonoids. The main components of phenolic and flavonoid compounds were ellagic acid, gallic acid, rutin, myricetin, and naringenin [75]. Greater amounts of caftaric acid, kaempferol and catechin were detected in Monkey pulp along with gallic acid, and epicatechin. In another study, Suleria et al. [38] detected several phenolic acids (caffeic acid, ferulic acid, *p*-coumaric acid, sinapinic acid) and flavonoids (quercectin-3-glucournoide, epicatechin gallate and kaempferol) in their study about fruit peels. Phenolics were detected in trace amounts in Ladyfinger and Cavendish pulp. In the same previous study, some tropical fruits, such as banana, custard apple, dragon fruit, papaya, passion fruit, and pineapple peels, did not contain high phenolic compounds [38]. Numerous studies have been undertaken to measure phenolic compounds in fruits; however, there is still a gap attributed to their complicated structures and the effect of environmental factors on secondary metabolite quantities between cultivars.

Our findings indicated that bananas contain a higher proportion of phenolic components with considerable antioxidant potential. Furthermore, quantitative investigation of key phenolic compounds in chosen cultivars using LC-ESI-QTOF-MS/MS may provide a broader knowledge of the association between phenolics, their structure, and their antioxidant capacity.

## 4. Conclusions

In conclusion, bananas contain a substantial number of phenolic compounds and have a high antioxidant potential. The results of FRAP, DPPH, ABTS, FICA, RPA, ^•^OH-RSA, and TAC revealed that banana peel and pulp have a good capacity for scavenging free radicals. Additionally, total phenolic content, total flavonoid content, and antioxidant capacity of banana cultivars varied. Our study identified 24 polyphenols using the sophisticated LC-ESI-QTOF-MS/MS analytical technology to identify and characterize polyphenols in banana. These findings indicate that banana peel has antioxidant potential and can be employed as an additive in the human food and animal feed markets for various health benefits. Due to the banana’s enhanced anti-radical properties, it is desirable to identify appropriate food applications for banana peel to provide value-added food items. Further investigation on the composition and antioxidant capacity of polyphenols extracted from banana peel and pulp is necessary for further understanding of their processes and include them into functional foods.

## Figures and Tables

**Table 1:** Polyphenol contents of six Australian grown bananas.

Banana Samples	TPC (mg GAE/g)	TFC (mg QE/g)	TTC (mg CE/g)
**Peels**			
Ripe Cavendish	0.54 ± 0.03 ^g–i^	0.01 ± 0.001 ^h–j^	0.19 ± 0.02 ^gh^
Unripe Cavendish	0.71 ± 0.04 ^d–f^	0.01 ± 0.0004 ^h–j^	0.5 ± 0.05 ^d–f^
Ripe Ladyfinger	0.56 ± 0.05 ^gh^	0.01 ± 0.00 ^g–i^	0.12 ± 0.01 ^hi^
Unripe Ladyfinger	1.15 ± 0.04 ^b^	0.007 ± 0.001 ^ij^	0.12 ± 0.01 ^hi^
Ripe Red Dacca	0.64 ± 0.05 ^e–g^	0.01 ± 0.0005 ^d–f^	0.38 ± 0.02 ^ef^
Ripe Plantain	0.87 ± 0.01 ^c^	0.03 ± 0.001 ^a^	0.53 ± 0.03 ^c–e^
Ripe Monkey	0.56 ± 0.03 ^gh^	0.006 ± 0.0004 ^j^	0.56 ± 0.01 ^cd^
Unripe Monkey	0.79 ± 0.05 ^cd^	0.013 ± 0.001 ^de^	0.44 ± 0.03 ^d–f^
Ripe Ducasse	1.32 ± 0.10 ^a^	0.02 ± 0.0004 ^b^	3.34 ± 0.2 ^a^
**Pulps**			
Ripe Cavendish	0.43 ± 0.01 ^h–j^	0.01 ± 0.0003 ^e–g^	0.02 ± 0.01 ^i^
Unripe Cavendish	0.55 ± 0.03 ^g–i^	0.01 ± 0.001 ^f–h^	0.66 ± 0.08 ^c^
Ripe Ladyfinger	0.42 ± 0.01 ^ij^	0.001 ± 0.0001 ^k^	0.42 ± 0.012 ^d–f^
Unripe Ladyfinger	0.76 ± 0.02 ^c–e^	0.01 ± 0.001 ^g–i^	1.52 ± 0.09 ^b^
Ripe Red Dacca	0.40 ± 0.01 ^j^	0.01 ± 0.0001 ^f–i^	-
Ripe Plantain	0.38 ± 0.01 ^j^	0.01 ± 0.001 ^d^	-
Ripe Monkey	0.43 ± 0.03 ^h–j^	0.02 ± 0.001 ^c^	0.001 ± 0.0 ^i^
Unripe Monkey	0.58 ± 0.03 ^fg^	0.02 ± 0.001 ^c^	0.02 ± 0.01 ^i^
Ripe Ducasse	1.28 ± 0.03 ^a^	0.03 ± 0.001 ^a^	0.34 ± 0.04 ^fg^

Values are mean ± standard deviation per gram fresh weight; *n* = 3 samples per sample. Values within the same column with different superscript letters (^a–k^) are significantly different from each other (*p* < 0.05). TPC (total phenolic contents); TFC (total flavonoid contents); TTC (total tannin contents); GAE (gallic acid equivalents); QE (quercetin equivalents); and CE (catechin equivalents).

**Table 2:** Antioxidant activity of six Australian grown banana.

Banana Samples	DPPH (mg AAE/g)	FRAP (mg AAE/g)	ABTS (mg AAE/g)	RPA (mg AAE/g)	FICA (mg EDTA/g)	^•^OH-RSA (mg AAE/g)	TAC (mg AAE/g)
**Peels**							
Ripe Cavendish	0.67 ± 0.02 ^d^	1.28 ± 0.06 ^de^	2.41 ± 0.19 ^a–c^	2.22 ± 0.23 ^de^	0.11 ± 0.01 ^e^	104.32 ± 1.72 ^ab^	0.12 ± 0.006 ^jk^
Unripe Cavendish	0.49 ± 0.01 ^ef^	1.77 ± 0.04 ^c^	2.29 ± 0.22 ^a–d^	2.26 ± 0.15 ^de^	0.04 ± 0.003 ^fg^	105.95 ± 0.72 ^a^	0.17 ± 0.005 ^ij^
Ripe Ladyfinger	0.59 ± 0.01 ^de^	1.04 ± 0.06 ^e–g^	2.69 ± 0.23 ^a^	6.15 ± 0.50 ^a^	0.38 ± 0.03 ^b^	103.60 ± 0.18 ^ab^	0.17 ± 0.01 ^ij^
Unripe Ladyfinger	0.47 ± 0.04 ^f^	1.35 ± 0.12 ^d^	2.49 ± 0.17 ^ab^	4.99 ± 0.04 ^b^	0.32 ± 0.02 ^c^	98.02 ± 1.57 ^cd^	0.13 ± 0.01 ^jk^
Ripe Red Dacca	0.13 ± 0.002 ^h^	0.87 ± 0.02 ^g–i^	1.76 ± 0.08 ^f–i^	1.76 ± 0.09 ^ef^	0.04 ± 0.0005 ^fg^	96.58 ± 1.72 ^de^	0.18 ± 0.01 ^h–j^
Ripe Plantain	0.66 ± 0.01 ^d^	1.20 ± 0.03 ^d–f^	2.17 ± 0.16 ^b–f^	2.37 ± 0.12 ^d^	0.04 ± 0.002 ^fg^	96.58 ± 0.36 ^de^	0.25 ± 0.01 ^gh^
Ripe Monkey	0.66 ± 0.02 ^d^	0.93 ± 0.09 ^gh^	0.59 ± 0.07 ^k^	2.36 ± 0.08 ^d^	0.59 ± 0.05 ^a^	96.04 ± 0.79 ^de^	0.24 ± 0.01 ^g–i^
Unripe Monkey	0.66 ± 0.01 ^d^	1.02 ± 0.10 ^e–g^	1.67 ± 0.09 ^g–i^	1.67 ± 0.17 ^fg^	0.12 ± 0.01 ^e^	95.14 ± 0.95 ^de^	0.12 ± 0.01 ^jk^
Ripe Ducasse	1.06 ± 0.09 ^b^	2.31 ± 0.10 ^b^	1.49 ± 0.13 ^hi^	4.33 ± 0.17 ^c^	0.26 ± 0.02 ^d^	106.67 ± 1.09 ^a^	0.08 ± 0.01 ^k^
**Pulps**							
Ripe Cavendish	0.13 ± 0.002 ^h^	0.69 ± 0.06 ^hi^	1.82 ± 0.16 ^e–h^	0.20 ± 0.05 ^i^	0.06 ± 0.002 ^f^	94.78 ± 0.48 ^de^	0.39 ± 0.03 ^e^
Unripe Cavendish	0.16 ± 0.01 ^h^	1.004 ± 0.02 ^fg^	2.20 ± 0.09 ^b–e^	1.71 ± 0.21 ^f^	0.05 ± 0.003 ^f^	103.06 ± 0.48 ^a–c^	0.58 ± 0.03 ^c^
Ripe Ladyfinger	0.26 ± 0.01 ^g^	0.86 ± 0.06 ^g–i^	1.77 ± 0.19 ^f–h^	-	-	99.46 ± 6.04 ^b–d^	0.27 ± 0.003 ^fg^
Unripe Ladyfinger	0.79 ± 0.05 ^c^	1.42 ± 0.05 ^d^	1.98 ± 0.14 ^c–g^	1.33 ± 0.06 ^f–h^	0.03 ± 0.002 ^fg^	97.48 ± 0.36 ^de^	1.03 ± 0.08 ^a^
Ripe Red Dacca	0.09 ± 0.01 ^h^	0.36 ± 0.02 ^j^	1.34 ± 0.07 ^ij^	0.89 ± 0.07 ^h^	0.02 ± 0.001 ^fg^	96.58 ± 1.18 ^de^	0.34 ± 0.004 ^ef^
Ripe Plantain	0.069 ± 0.004 ^h^	0.62 ± 0.01 ^ij^	2.35 ± 0.05 ^a–c^	1.12 ± 0.03 ^h^	0.06 ± 0.005 ^f^	97.12 ± 1.003 ^de^	0.29 ± 0.003 ^fg^
Ripe Monkey	0.26 ± 0.01 ^g^	0.80 ± 0.02 ^g–i^	0.98 ± 0.05 ^jk^	0.84 ± 0.07 ^h^	0.05 ± 0.003 ^f^	94.96 ± 0.65 ^de^	0.40 ± 0.017 ^de^
Unripe Monkey	0.32 ± 0.01 ^g^	0.87 ± 0.05 ^g–i^	1.91 ± 0.06 ^d–h^	1.18 ± 0.10 ^gh^	0.06 ± 0.002 ^f^	92.25 ± 0.65 ^e^	0.47 ± 0.03 ^d^
Ripe Ducasse	1.68 ± 0.06 ^a^	2.85 ± 0.28 ^a^	1.86 ± 0.14 ^d–h^	5.67 ± 0.04 ^a^	0.04 ± 0.003 ^fg^	106.85 ± 1.77 ^a^	0.69 ± 0.04 ^b^

Values are mean ± standard deviation per gram fresh weight; *n* = 3 samples per sample. Values within the same column with different superscript letters (^a–k^) are significantly different from each other (*p* < 0.05). DPPH (2,2′-diphenyl-1-picrylhydrazyl assay); FRAP (ferric reducing antioxidant power assay); ABTS (2,2′-azino-bis-3-ethylbenzothiazoline-6-sulfonic acid assay); TAC (total antioxidant capacity); AAE (ascorbic acid equivalents); RPA (reducing power assay); FICA (ferrous ion chelating activity); ^•^OH-RSA (hydroxyl-radical scavenging activity); and EDTA (ethylenediaminetetraacetic acid).

**Table 3:** Pearson’s linear correlation between banana phenolic content and their antioxidant capacity.

Variables	TPC	TFC	TTC	DPPH	FRAP	ABTS	RPA	FICA	^•^OH-RSA
TFC	0.599 **								
TTC	0.575 **	0.286							
DPPH	0.790 **	0.584 **	0.444						
FRAP	0.859 **	0.564 *	0.539 *	0.896 **					
ABTS	0.107	−0.040	−0.161	−0.010	0.138				
RPA	0.706 **	0.360	0.227	0.670	0.659 **	0.338			
FICA	0.182	−0.197	0.160	0.208	0.053	−0.216	0.513 *		
^•^OH-RSA	0.466 *	0.183	0.417	0.563 *	0.736 **	0.362	0.603 **	0.069	
TAC	−0.016	0.136	0.033	0.187	0.136	−0.050	−0.171	−0.381	−0.094

* Significant correlation at *p* ≤ 0.05; ** significant correlation at *p* ≤ 0.01.

**Table 4:** Characterization of phenolic compounds from different banana cultivars by LC-ESI-QTOF-MS/MS.

No.	Proposed Compounds	Molecular Formula	RT (min)	Ionization (ESI+/ESI−)	Molecular Weight	Theoretical (*m/z*)	Observed (*m/z*)	Error (ppm)	MS^2^ Product ions	Sample Code
	**Phenolic acid**									
	**Hydroxyphenylpropanoic acids**									
**1**	3-Hydroxyphenylpropionic acid	C_9_H_10_O_3_	30.544	[M–H]^–^	166.0630	165.0557	165.0556	−0.6	165, 121, 119	RCPl
	**Hydroxycinnamic acids**									
**2**	Ferulic acid	C_10_H_10_O_4_	9.105	[M–H]^–^	194.0579	193.0506	193.0506	0.0	178, 149, 134	RCP
**3**	Caffeic acid	C_9_H_8_O_4_	41.086	[M–H]^–^	180.0423	179.035	179.035	0.0	143, 133	UCP
**4**	*p*-Coumaroyl glycolic acid	C_11_H_10_O_5_	62.765	[M+H]^+^	222.0528	223.0601	223.0599	−0.9	163	* RCPl, ULPl, RRPl, RPPl, RMP, RDPl
	**Hydroxyphenylacetic acids**									
**5**	3,4-Dihydroxyphenylacetic acid	C_8_H_8_O_4_	20.831	[M–H]^–^	168.0423	167.035	167.0341	−5.4	149, 123	* RRPl, UCPl
	**Hydroxybenzoic acids**									
**6**	3,4-*O*-Dimethylgallic acid	C_9_H_10_O_5_	5.557	[M+H]^+^	198.0528	199.0601	199.0595	−3.0	153, 139, 125, 111	RLPl
	**Flavonoids**									
	**Anthocyanins**									
**7**	Delphinidin 3-*O*-(6’’-acetyl-galactoside)	C_23_H_23_O_13_	82.037	[M–H]^–^	507.1139	506.1066	506.1066	0.0	303, 507	* RPPl, UCPl, UCP, RLP, ULP, UMPl, UMP, RMPl, RMP
**8**	Pelargonidin 3-*O*-(6’’-succinyl-glucoside)	C_25_H_25_O_13_	40.051	[M–H]^–^	533.1295	532.1222	532.1181	−7.7	533, 473, 443, 383, 353	UCP
**9**	Cyanidin 3,5-*O*-diglucoside	C_27_H_31_O_16_	89.507	[M+H]^+^	611.1612	612.1685	612.1674	−1.8	449, 287	* RCP, RRPl
**10**	Malvidin 3-*O*-(6’’-acetyl-glucoside)	C_25_H_27_O_13_	6.241	[M+H]^+^	535.1452	536.1525	536.1525	0.0	535, 481, 463, 445	RRPl
	**Isoflavonoids**									
**11**	2’-Hydroxyformononetin	C_16_H_12_O_5_	24.296	[M+H]^+^	284.0685	285.0758	285.0755	−1.1	270, 229	RMP
	**Flavonols**									
**12**	Isorhamnetin 3-*O*-glucoside 7-*O*-rhamnoside	C_28_H_32_O_16_	29.971	[M–H]^–^	624.169	623.1617	623.1622	0.8	433, 315, 300, 271	UCPl
**13**	Myricetin 3-*O*-rutinoside	C_27_H_30_O_17_	24.472	[M–H]^–^	626.1483	625.141	625.1397	−2.1	301	UCPl
**14**	Patuletin 3-*O*-glucosyl-(1->6)-[apiosyl(1->2)]-glucoside	C_33_H_40_O_22_	37.955	[M–H]^–^	788.2011	787.1938	787.1918	−2.5	625, 463, 301, 271	RRPl
**15**	Quercetin 3-*O*-xylosyl-glucuronide	C_26_H_26_O_17_	38.624	[M+H]^+^	610.117	611.1243	611.1238	−0.8	479, 303, 285, 239	UCPl
	**Flavanols**									
**16**	(+)-Gallocatechin 3-*O*-gallate	C_22_H_18_O_11_	3.075	[M–H]^–^	458.0849	457.0776	457.0773	−0.7	305, 169	RRPl
	**Flavones**									
**17**	Apigenin 7-*O*-apiosyl-glucoside	C_26_H_28_O_14_	88.42	[M–H]^–^	564.1479	563.1406	563.1402	−0.7	503, 443, 383, 353, 325, 297	UCP
**18**	Chrysoeriol 7-*O*-glucoside	C_22_H_22_O_11_	89.788	[M+H]^+^	462.1162	463.1235	463.1224	−2.4	445, 427, 409, 381	RCPl
	**Flavanones**									
**19**	Neoeriocitrin	C_27_H_32_O_15_	27.305	[M–H]^–^	596.1741	595.1668	595.1696	4.7	431, 287	RRPl
	**Other polyphenols**									
	**Hydroxycoumarins**									
**20**	Scopoletin	C_10_H_8_O_4_	7.392	[M–H]^–^	192.0423	191.035	191.0349	−0.5	176	* RPPl, RCP, UCP
**21**	Urolithin A	C_13_H_8_O_4_	4.563	[M–H]^–^	228.0423	227.035	227.035	0.0	198, 182	UCPl
**22**	Umbelliferone	C_9_H_6_O_3_	35.668	** [M+H]^+^	162.0317	163.039	163.0387	−1.8	145, 135, 119	* RMPl, UCP
	**Hydroxyphenylpropenes**									
**23**	2’-Hydroxyformononetin	C_16_H_12_O_5_	24.296	[M+H]^+^	284.0685	285.0758	285.0755	−1.1	270, 229	RMP
	**Furanocoumarins**									
**24**	Isopimpinellin	C_13_H_10_O_5_	5.61	[M+H]^+^	246.0528	247.0601	247.0583	−7.3	232, 217, 205, 203	ULP

* = compound detected in more than one sample, with data presented only asterisk, ** = compound found in both positive [M+H]^+^ and negative modes [M–H]^–^. RT stands for “retention time”. Bananas were presented with abbreviations. Ripe Cavendish peel (RCPl), Ripe Cavendish pulp (RCP), Unripe Cavendish peel (UCPl), Unripe Cavendish pulp (UCP), Unripe Ladyfinger peel (ULPl), Ripe red dacca peel (RRPl), Ripe Plantain peel (RPPl), Ripe Monkey pulp (RMP), Ripe Ducasse peel (RDPl), Ripe Ladyfinger peel (RLPl), Ripe Ladyfinger pulp (RLP), Unripe Ladyfinger pulp (ULP), Unripe Monkey peel (UMPl), Unripe Monkey pulp (UMP), and Ripe Monkey peel (RMPl).

## Data Availability

The data presented in this study are available in this manuscript.
